# The prevalence of pica in tuberous sclerosis complex

**DOI:** 10.1186/s40064-015-0841-x

**Published:** 2015-02-01

**Authors:** Patrick J Morrison, Tara O’Neill, Rachel Hardy, Charles W Shepherd, Deirdre E Donnelly

**Affiliations:** Department of Genetic Medicine, Belfast HSC Trust, Lisburn Road, Belfast, BT9 7AB UK; Centre for Cancer Research and Cell Biology, Queens University of Belfast, 97 Lisburn Road, Belfast, BT9 7AE UK

**Keywords:** Pica, Tuberous sclerosis, Northern Ireland population

## Abstract

**Background:**

Pica and Tuberous sclerosis complex (TSC) are rare disorders. We carried out a population survey of pica in our TSC patient population.

**Findings:**

Pica was identified in four percent of cases of TSC. It was associated with adult onset or persistence into adulthood, epilepsy, severe learning difficulties and anaemia.

**Conclusions:**

Pica in TSC is a rare disorder and a coherent history may be difficult to obtain from patients. The prevalence of pica is likely to be underdiagnosed. Pica is a recognised feature in adults with TSC and prompt recognition of this disorder should allow better management of patients with TSC.

## Findings

Pica - the persistent ingestion of non- nutritive substances for greater than 1 month at an age at which this is developmentally inappropriate - is a rare disorder. The true incidence is unknown but it occurs frequently in childhood, but apart from pregnancy is rare in adulthood (Hagopian et al. 2011). Disorders causing autism may have pica as a recognised feature mainly between 10 and 20 years of age (Hagopian et al. 2011). Tuberous sclerosis complex (TSC) is a rare autosomal dominant neurogenetic disorder with a combination of features including epilepsy, hypopigmented macules of the skin and other dermatological findings, variable learning disability, autistic tendencies and angiomyolipomas of the kidney (Morrison et al. 2010; Hardy et al. 2012; Morrison and Ryan 2012; Morrison 2009).

The prevalence of pica in TSC cases has not previously been documented. We investigated a population of TSC to see if pica is a recognised feature of tuberous sclerosis and determine its prevalence, age at onset and any potential associated recognisable features.

### Methods

A register of cases of TSC has been maintained for the Northern Ireland population since 1967. All cases are seen centrally at a regional TSC clinic. 100 cases were alive on 1st January 2014. An analysis of case records from the TSC clinical register for the Northern Ireland population (Devlin et al. 2006) was carried out. Four cases of pica were identified out of the 100 recorded living TSC cases. The clinical features are shown in Table 1. The onset of pica was determined from parents of the cases.

**Table 1:** **Clinical features of the pica cases**

	**Gender**	**Pica onset**	**Current age**	**TSC mutation**	**Learning difficulty**	**Autistic features**	Haemoglobin at onset*	**Epilepsy**	**Pica item**
Case 1	Female	41	47	Not known	Severe	Yes	110 g/l	Yes	Cigarette butts
Case 2	Male	41	46	TSC2	Severe	Yes	Not known	Yes	Furniture
Case 3	Female	14	18	TSC1	Moderate	Yes	59 g/l	Yes	Furniture, cleaning implements
Case 4	Male	4	47	TSC2	Severe	Yes	82 g/l	Yes	Furniture

*Normal laboratory range 120–150 g/l.

This research was carried out according to our institution’s (Belfast HSC Trust) guidelines. Permission was granted to access all relevant patient data.

### Results

Four percent of cases of TSC (4 out of 100 cases alive on prevalence day) were documented to have pica. At least three of the cases appeared to have associated anaemia and in two of these (cases 3 and 4) the pica resolved within two months of the anaemia being treated based on reports from parents that the pica had stopped. All four cases of TSC had severe learning difficulties, epilepsy and autistic features categorised by developmental paediatric assessment. Three were adults although one of these had developed pica aged four and it was of long standing. Furniture was ingested (rather than just licking or chewing) and in one case the inside foam of a settee was ingested.

### Discussion

Both TSC and Pica are rare and under-recognised disorders. Pica in TSC was first reported by Torii in 1971 in a Japanese adolescent male with TSC epilepsy and learning difficulties (Torii et al. 1971). No reports have been identified subsequently and the prevalence is unknown. The fact that pica was present in four percent of cases all associated with learning difficulties and epilepsy suggests that this is a recognised behavioural feature of tuberous sclerosis and may reflect neuronal change caused by cortical tubers causing epilepsy and the features may be worsened by an underlying anaemia. There was no direct correlation with either TSC1 or TSC2 mutations. The age of onset and duration of pica in TSC may be different from transient pica associated with childhood or with classical pica in autism with the onset in cases of TSC being more variable and more sustained in duration, however this sample size is small and further work in this area would be helpful to clarify any potential differences. The association with anaemia is interesting and the fact that it resolved after treatment of the anaemia may be important. There is no clear association between anaemia and pica documented in the literature in this group although the sample size is small so no clear conclusions can be drawn, but this could be an important area of interest and checking the haemoglobin in cases of pica may be helpful.

### Conclusions

The minimum prevalence of pica in tuberous sclerosis in the Northern Ireland population is four percent. Pica should be looked for in cases of TSC particularly with adult cases with epilepsy and severe learning difficulties, as a coherent confirmatory history may be difficult to obtain. Patients should be assessed for anaemia as any underlying anaemia should be treated promptly and may improve the pica.
